# Intact Imaging of Human Heart Structure Using X-ray Phase-Contrast Tomography

**DOI:** 10.1007/s00246-016-1527-z

**Published:** 2016-11-30

**Authors:** Yukihiro Kaneko, Gen Shinohara, Masato Hoshino, Hiroyuki Morishita, Kiyozo Morita, Yoshihiro Oshima, Masashi Takahashi, Naoto Yagi, Yutaka Okita, Takuro Tsukube

**Affiliations:** 1 Division of Cardiovascular Surgery, National Medical Center for Children and Mothers, 2-10-1, Okura, Setagaya-ku, Tokyo, 157-8535 Japan; 2 Department of Cardiovascular Surgery, Jikei University School of Medicine, Tokyo, Japan; 3 Japan Synchrotron Radiation Research Institute (SPring-8), Sayo, Japan; 4 Department of Cardiovascular Surgery, Kobe Children’s Hospital, Kobe, Japan; 5 Division of Thoracic and Cardiovascular Surgery, Niigata University Graduate School of Medical and Dental Sciences, Niigata, Japan; 6 Division of Cardiovascular Surgery, Graduate School of Medicine, Kobe University, Kobe, Japan; 7 Division of Cardiovascular Surgery, Japanese Red Cross Kobe Hospital, Kobe, Japan

**Keywords:** Heart defects, Congenital, Synchrotrons, Microscopy, Phase-contrast, Heart conduction system

## Abstract

Structural examination of human heart specimens at the microscopic level is a prerequisite for understanding congenital heart diseases. It is desirable not to destroy or alter the properties of such specimens because of their scarcity. However, many of the currently available imaging techniques either destroy the specimen through sectioning or alter the chemical and mechanical properties of the specimen through staining and contrast agent injection. As a result, subsequent studies may not be possible. X-ray phase-contrast tomography is an imaging modality for biological soft tissues that does not destroy or alter the properties of the specimen. The feasibility of X-ray phase-contrast tomography for the structural examination of heart specimens was tested using infantile and fetal heart specimens without congenital diseases. X-ray phase-contrast tomography was carried out at the SPring-8 synchrotron radiation facility using the Talbot grating interferometer at the bending magnet beamline BL20B2 to visualize the structure of five non-pretreated whole heart specimens obtained by autopsy. High-resolution, three-dimensional images were obtained for all specimens. The images clearly showed the myocardial structure, coronary vessels, and conduction bundle. X-ray phase-contrast tomography allows high-resolution, three-dimensional imaging of human heart specimens. Intact imaging using X-ray phase-contrast tomography can contribute to further structural investigation of heart specimens with congenital heart diseases.

**Electronic supplementary material:**

The online version of this article (doi:10.1007/s00246-016-1527-z) contains supplementary material, which is available to authorized users.

## Introduction

Examination of the structure of human heart specimens at the microscopic level is a prerequisite for understanding congenital heart diseases. Due to the scarcity of heart specimens with complex heart diseases, it is important not to destroy or alter the property of the specimens during examination.

X-ray phase-contrast tomography (XPCT) is an imaging modality for soft tissue specimens that does not destroy or alter the properties of the specimen [1–3]. The structures of soft tissue specimens, including mouse fetuses, porcine eyes, and the human aortic wall, have been clearly visualized using XPCT [4–6]. We tested the feasibility of XPCT for the structural investigation of heart specimens using infantile and fetal hearts without congenital diseases.

## Methods

### Specimens

Human heart specimens obtained by autopsy from two fetuses and three infants were used. Absence of congenital heart disease was confirmed at autopsy. The ethical committees of the participating hospitals approved the study and waived the requirement of consent from the family of the deceased.

### X-ray Phase-Contrast Tomography

The XPCT was carried out at the SPring-8 synchrotron radiation facility using the Talbot grating interferometer at the bending magnet beamline BL20B2 to visualize the structure of heart specimens. The XPCT system has been described in detail elsewhere [4, 5]. Briefly, the system was located 200 m from the synchrotron X-ray source. The X-ray energy was tuned to 25 keV by passing through a Si (111) double-crystal monochromator. The specimen was placed in an appropriately sized saline-filled plastic canister and rotated slowly in the canister with a rotating stage. The Talbot grating interferometer consisting of a phase grating and an absorption grating was placed behind the specimen. Moiré fringes generated by the interferometer were detected by an X-ray detector. Phase retrieval was attained using a five-step phase-stepping procedure by shifting the absorption grating iteratively with a Piezo-driven stage. The data were processed by a high-throughput system to create a three-dimensional image. The system specifications were as follows: field of view, 24.7 mm (H) × 17.1 mm (V); voxel size, 12.5 μm; target density range, 0.9–1.2 g/cm^3^; density resolution, 1 mg/cm^3^; exposure time, 1 s/image; number of projections, 900; radiation dose 75 Gy. The horizontal field of view, namely the field of view in the reconstructed slice image, can be extended up to 40 mm by employing an offset tomographic scan.

## Results

**Fig. 1:**
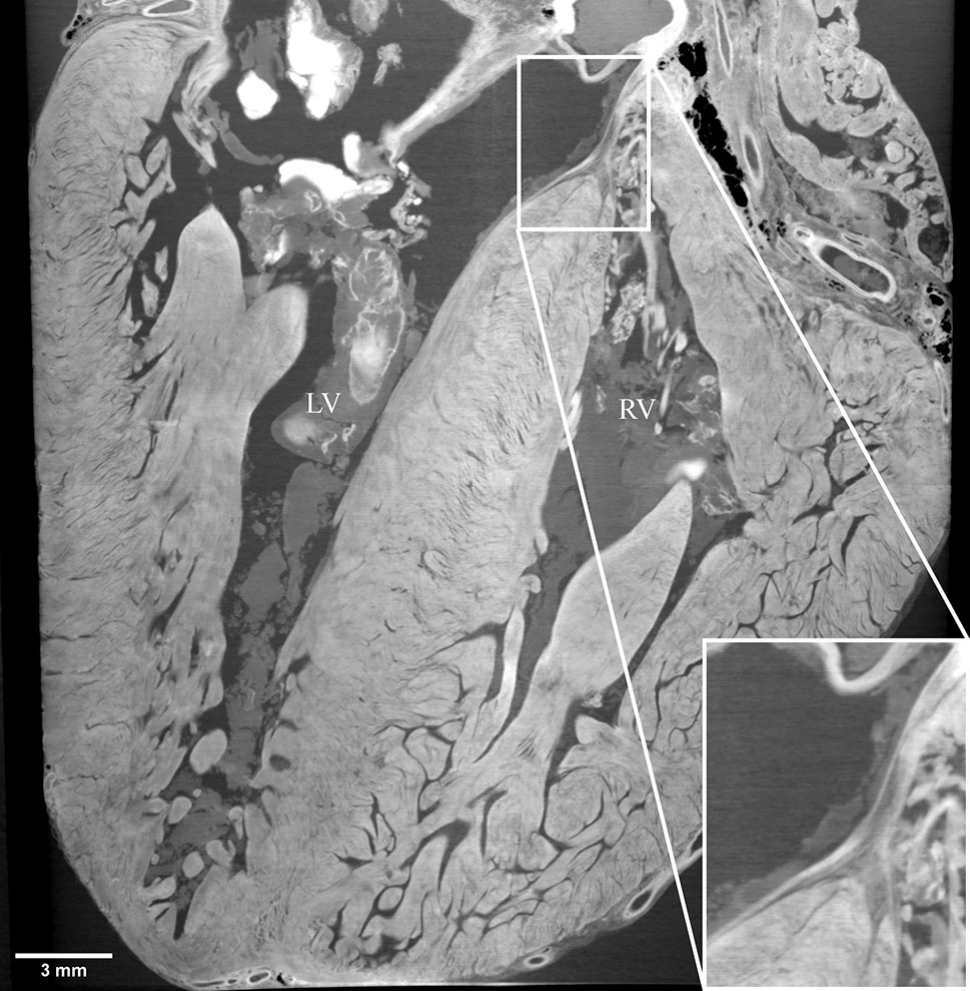
XPCT image showing the conduction bundle. Two-dimensional X-ray phase-contrast tomography (XPCT) image showing the ventricular septum of a fetal heart. Enlarged view of the crest of the ventricular septum shows *dark* inverted Y-shaped structure indicating the atrioventricular bundle dividing into *left* and *right* bundle branches. *LV* left ventricle; *RV* right ventricle

**Fig. 2:**
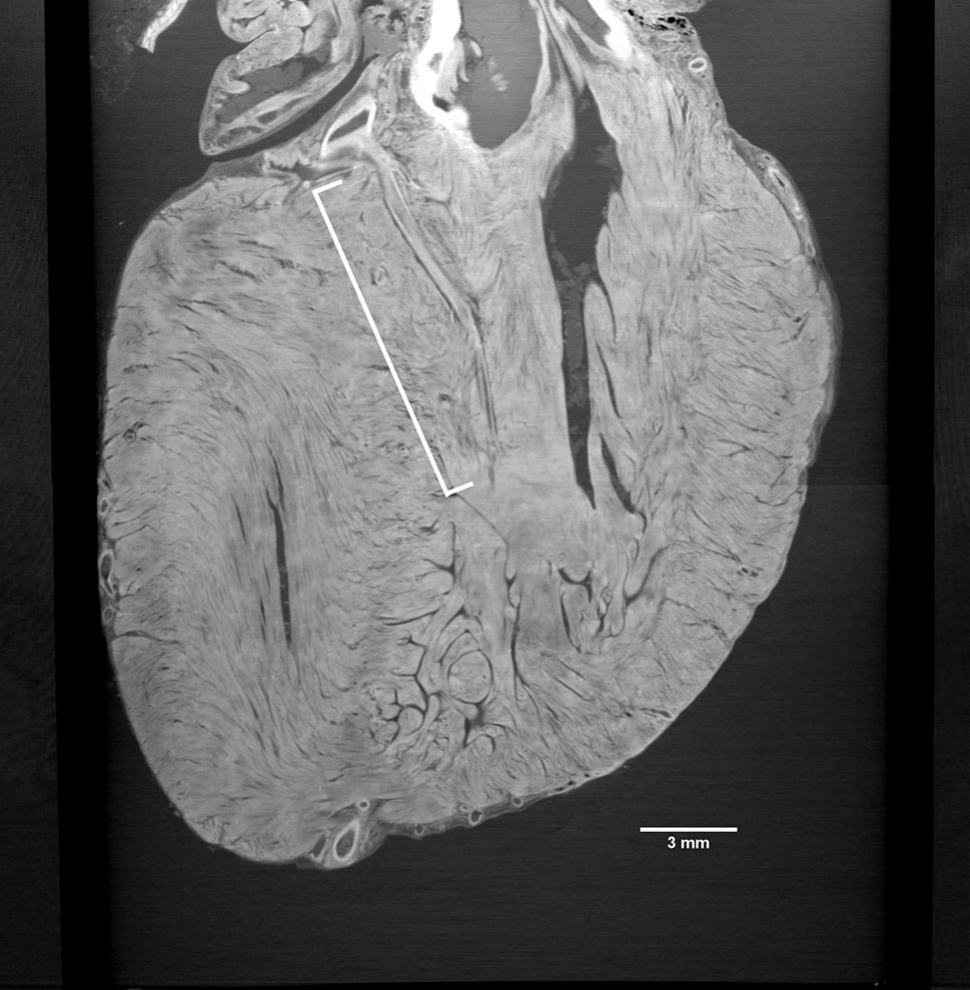
XPCT image showing the first septal branch of the left anterior descending artery. Two-dimensional XPCT image of the same heart at a different angle showing the first septal branch (*white bracket*) originating from the left anterior descending artery

High-resolution images were obtained for all specimens. A two-dimensional slice showing the ventricular septum of a fetal heart is shown in Fig. 1. The figure clearly shows the myocardial structure and displays an inverted Y-shaped dark structure at the crest of the ventricular septum indicating the conduction bundle. The conduction bundle contains more fat tissues and appears darker in XPCT than the neighboring myocardium [7]. A video containing this slice is provided as Supplementary video 1. Figure 2 shows the left anterior descending artery and its first septal branch in the same heart. A video containing this slice is provided as Supplementary video 2. A three-dimensional volume-rendered reconstruction of the same specimen with special attention paid to the ventricular septum is provided as Supplementary video 3.

### Comments

We have shown that XPCT provides high-resolution, three-dimensional images without destroying or altering the property of human heart specimens.

### Alternative Imaging Modalities

Other modalities for visualizing the structures of soft tissues include histological observation, absorption contrast computed tomography (ACCT), and magnetic resonance imaging (MRI). However, each of these modalities has its shortcomings.

Histological light microscopic observation is the standard technique for structural investigation, but a major limitation of this technique is the destruction of the specimen by sectioning, which renders the specimen unusable for other studies. ACCT has a high spatial resolution of up to 0.7 μm [8]; however, its contrast sensitivity in imaging soft tissues is about 1000 times poorer compared with XPCT [9]. Remedies for low contrast sensitivity include staining and injection of contrast agent into the blood vessels. However, the use of stains such as osmium, inorganic iodine, and phosphotungstic acid may alter the chemical and mechanical properties of the samples, and hence precluding subsequent studies. Furthermore, the homogeneous injection of contrast agent into the heart postautopsy is technically difficult because the heart chambers have already been incised for macroscopic investigation. Consequently, intact imaging of human heart specimens by ACCT is impractical. MRI may be used for intact soft tissue imaging; however, its spatial resolution of about 40 μm yields less detailed images compared with XPCT.

### Alternative XPCT Techniques

Several XPCT imaging techniques have been developed, including the propagation-based technique, analyzer-based technique, crystal interferometry, grating interferometry, and the grating non-interferometric technique. We adopted grating interferometry because it provides adequate field of view, as well as high-density sensitivity and a dynamic range, and does not suffer from interferometer drift. A comprehensive review of each technique can be found elsewhere [10].

In our current XPCT imaging system, investigation of the whole adult human heart is not possible due to two limitations. The first limitation is unsatisfactory spatial resolution. The size of the specimen is limited by the XPCT system’s effective field of view, which is defined by the X-ray detector, grating area size, and X-ray beam size. Since the X-ray detector is composed of a digital camera with pixel array, the field of view is defined by the pixel size and number of pixels. This means that a larger pixel size makes it possible to obtain a larger field of view. However, a larger pixel size decreases the quality of spatial resolution. The height of the specimen is limited to 30 mm due to the X-ray beam size of our current XPCT imaging system. It may be possible to measure a larger specimen up to 100 mm in width using an X-ray detector with a larger field of view and a larger grating area size at the expense of spatial resolution. The second limitation is increased X-ray absorption. A larger specimen must be placed in a larger saline-filled canister for XPCT imaging. If we use a canister of 100 mm in thickness, the X-rays irradiated into the canister are mostly absorbed by saline and the specimen. It would be necessary to use higher X-ray energy for X-ray transmission when imaging a specimen as large as 100 mm in diameter.

### Future Clinical Contribution

In spite of extensive research, the structure of hearts with congenital heart diseases has not been elucidated. For example, a heart with isomerism may have anterior atrioventricular bundle, posterior atrioventricular bundle, or twin atrioventricular sling. The location of the conduction bundle cannot be definitively predicted in an isomeric heart [11]. Furthermore, definitive imaging criteria to distinguish myocardial non-compaction and dilated cardiomyopathy have yet to be established [12]. Surgical injury to the coronary arterial branches supplying the conduction bundle is postulated to induce heart block. The clinical impact of coronary arterial branch injury from myocardial resection or ventricular septal defect enlargement remains unclear [13]. Using XPCT, we will examine as many heart specimens with congenital heart diseases as possible. The structures of the conduction tissues, cardiac musculature, and coronary vessels, as well as their relationships, can be easily studied. We believe that XPCT is a powerful tool for elucidating unanswered questions concerning congenital heart diseases.

Clinical application of XPCT is gaining traction [14]; however, imaging of the in vivo heart is challenging as it is surrounded by the air-filled alveoli of the lungs, which cause random refraction of irradiated X-rays. Moreover, the ribs absorb X-rays strongly. As a result, detection of phase-shift values inside the heart is more difficult compared with using an isolated heart. Future development of the XPCT system for in vivo human heart imaging can be expected.

## Conclusion

Due to the scarcity of heart specimens with congenital diseases, imaging modalities that allow examination without destroying or altering the property of the specimens are desirable. XPCT was feasible for high-resolution, three-dimensional imaging of human heart specimens. Intact imaging using XPCT will expand our understanding of congenital heart diseases.

## Electronic supplementary material

Below is the link to the electronic supplementary material.

Supplementary video 1 Video containing the slice shown in Fig. 1 (MP4 16049 kb)

Supplementary video 2 Video containing the slice shown in Fig. 2 (MP4 9816 kb)

Supplementary video 3 Three-dimensional volume-rendered reconstruction of the same heart. The heart valves, subvalvular apparatus, ventricular septum, and myocardial morphology are clearly depicted. The conduction bundle is visualized at the elapsed time of 41 s (MP4 20223 kb)
